# Simultaneous overexpression of three enzymes of chloroplast metabolism fails to improve CO_2_ assimilation or biomass

**DOI:** 10.1093/jxb/erag121

**Published:** 2026-03-07

**Authors:** Pauline Lemonnier, Shellie Wall, Hussein Gherli, Beatriz Moreno-Garcia, Chidi Afamefule, Tracy Lawson, Christine A Raines, Patricia E Lopez-Calcagno

**Affiliations:** School of Life Sciences, University of Essex, Wivenhoe Park, Colchester CO4 3SQ, UK; School of Life Sciences, University of Essex, Wivenhoe Park, Colchester CO4 3SQ, UK; School of Life Sciences, University of Essex, Wivenhoe Park, Colchester CO4 3SQ, UK; School of Life Sciences, University of Essex, Wivenhoe Park, Colchester CO4 3SQ, UK; School of Life Sciences, University of Essex, Wivenhoe Park, Colchester CO4 3SQ, UK; School of Life Sciences, University of Essex, Wivenhoe Park, Colchester CO4 3SQ, UK; School of Life Sciences, University of Essex, Wivenhoe Park, Colchester CO4 3SQ, UK; School of Natural and Environmental Sciences, Newcastle University, Newcastle NE1 7RU, UK; Max Planck Institute for Molecular Plant Physiology, Germany

**Keywords:** ADP-glucose pyrophosphorylase, carbon assimilation, crop productivity, fructose-1,6-bisphosphate aldolase, model-informed genetic manipulation, *Nicotiana tabacum*, photosynthesis optimization, sedoheptulose-1,7-bisphosphatase, sink capacity, yield improvement

## Abstract

Ensuring an adequate food supply amidst a growing global population and climate change challenges necessitates innovative strategies to enhance crop productivity. Previous studies have demonstrated that the simultaneous stimulation of different photosynthesis-related processes can increase the rate of photosynthetic carbon assimilation and plant biomass. This study evaluates an approach based on modelling aimed at simultaneously increasing photosynthetic and sink capacities in *Nicotiana tabacum* by overexpressing three key enzymes: sedoheptulose-1,7-bisphosphatase (SBPase), fructose-1,6-bisphosphate aldolase (FBP aldolase), and ADP-glucose pyrophosphorylase (AGPase). Our results showed that this strategy does not significantly improve growth or carbon assimilation in *Nicotiana tabacum* under the tested conditions. This suggests that while the model informing our work offers a valuable framework, its application may require adjustments based on species and environmental conditions. Future research should explore these genetic modifications in species with larger sink capacities and under a range of growth conditions to fully realize the potential of photosynthetic optimization.

## Teaser

Model-informed overexpression of SBPase, FBP aldolase, and AGPase in *Nicotiana tabacum* did not enhance carbon assimilation or biomass, providing insights into species-specific responses for optimizing carbon assimilation and biomass production.

## Introduction

As our population continues to increase, ensuring an adequate food supply becomes increasingly challenging. To secure future food supplies without increasing current agricultural lands, in a world affected by climate change, the continued development of innovative strategies to enhance crop productivity is needed. Central to this challenge are the processes of photosynthesis and the intricate source–sink relations that regulate carbon allocation within plants. Understanding how we can optimize these processes has the potential to lead to significant improvements in agricultural output.

Over the last two decades, a number of empirical studies have shown consistently how increasing the investment in some Calvin–Benson–Bassham (CBB) cycle enzymes (by their overexpression) is able to increase photosynthetic productivity. The first efforts focused on manipulation of single enzymes, a good example of which is the overexpression of sedoheptulose-1,7-bisphosphatase (SBPase) in four different species, *Arabidopsis thaliana* (Simkin *et al.*, 2017), tobacco (Lefebvre *et al.*, 2005; Rosenthal *et al.*, 2011; Simkin *et al.*, 2015), tomato (Ding *et al.*, 2016), and wheat (Driever *et al.*, 2017), which consistently showed increases in both photosynthetic carbon assimilation and yield. Other targets, such as the introduction of the cyanobacterial fructose-1,6-bisphosphatase/sedoheptulose-1,7-bisphosphatase (cyFBP/SBPase) enzyme (Miyagawa *et al.*, 2001; Tamoi *et al.*, 2006; Ichikawa *et al.*, 2010; Köhler *et al.*, 2017), and the overexpression of the chloroplast fructose-1,6-bisphosphate aldolase (FBP aldolase) (Uematsu *et al.*, 2012; Simkin *et al.*, 2015, 2017; Cai *et al.*, 2022) have also shown increases in productivity. However, more recently, work focused on the simultaneous stimulation of multiple enzymes has shown increased success in photosynthesis stimulation. Simkin *et al.* (2015, 2017) showed how the combined overexpression of SBPase and FBP aldolase in *A. thaliana* and tobacco led to further increases in productivity compared with the single overexpression of either of these enzymes. Furthermore, these studies and others have also shown a positive effect on photosynthetic carbon assimilation and yield by not only stimulating the CBB cycle but, when combining this with targets in other photosynthetic processes such as photorespiration, by increasing the levels of the H-protein of the glycine cleavage system (Simkin *et al.*, 2017); the electron transport chain, by introducing cytochrome *c*_6_ in higher plants (Lopez-Calcagno *et al.*, 2020); or by combination with the putative inorganic carbon transporter B (ictB) (Simkin *et al.*, 2015).

In agreement with these pieces of empirical evidence and using a modelling approach, Zhu *et al.* (2007) proposed that ‘the distribution of resources between enzymes of photosynthetic carbon metabolism might be assumed to have been optimized by natural selection. However, natural selection for survival and fecundity does not necessarily select for maximal photosynthetic productivity’. Taking this into account, together with the rapid changes to atmospheric CO_2_ concentrations which we are currently experiencing, calls for a review of the enzyme balance for maximizing the light-saturated rate of photosynthesis. The authors proposed that currently plants have an overinvestment in photorespiratory pathway (PCOP) enzymes and underinvestment in CBB cycle enzymes such as Rubisco, SBPase, and FBP aldolase. Furthermore, they proposed that an increase in sink capacity, such as that obtained by increasing ADP-glucose pyrophosphorylase (AGPase), would also be needed for an increased CO_2_ uptake rate. Specifically, the model included 23 enzymes and proposed that for optimizing photosynthesis at around current atmospheric CO_2_ concentrations, the levels of seven of these enzymes, all belonging to the CBB cycle with the exception of AGPase, would need to be increased, while the levels of the 14 other enzymes, two of which belong to the CBB cycle and seven to the PCOP, would need to be decreased.

In addition to this proposal (Zhu *et al.*, 2007), there is also empirical evidence that the limitation on P_i_ recycling due to conditions where the potential for photosynthesis exceeds the rate of end-product synthesis, such as limitations in the capacity for sucrose and starch synthesis (sink), can induce a negative feedback response in net photosynthesis (Paul and Foyer, 2001; Pieters *et al.*, 2001; Paul and Pellny, 2003). Evidence of the role of leaf starch in influencing the capacity for photosynthesis has clearly been shown using starch-deficient and starch-null Arabidopsis mutants. In these plants, the rates of CO_2_ assimilation and O_2_ evolution were proportional to the level of AGPase; being highest for the wild type, and lowest for the starch-null mutants (Sun *et al.*, 1999). This strongly suggested that leaf starch is important not only as a transient reserve whose metabolism supports heterotrophic growth in the dark, or as a transient sink, but also as an essential end-product in triose-phosphate utilization (Gibson *et al.*, 2011).

With this in mind, we have taken a ‘minimal approach’ to simultaneously increase photosynthetic and sink capacity in *Nicotiana tabacum*. Given the strong empirical evidence that down-regulating enzymes of the PCOP can have a negative effect in photosynthesis and plant growth (Bauwe, 2023) and that overexpression of certain enzymes in the CBB cycle can lead to significant increases in photosynthesis and plant growth (Uematsu *et al.*, 2012; Simkin *et al.*, 2015, 2017; Cai *et al.*, 2022), we have focused on overexpression of the three enzymes modelled to require the largest increases: SBPase, chloroplast FBP aldolase (referred from hereon as FBP aldolase), and AGPase. This paper covers the main findings of these experiments and suggests further considerations for the success of approaches trying to optimize photosynthesis manipulation with source–sink relationships.

## Materials and methods

### Assembly of a binary construct for simultaneous overexpression of SBPase, FBP aldolase, and AGPase

The coding sequences—including native chloroplast transit peptides—for SBPase (AT3G55800) and AGPase large subunit 1 (AT5G19220) from *A. thaliana*, and FBP aldolase (NM_001346974) from *Solanum lycopersicum* were domesticated for use in Golden Gate cloning (Engler *et al.*, 2009, 2014; Weber *et al.*, 2011), synthesized as level 0 modules, and used in the generation of the SFA construct via Golden Gate cloning. The transgenes were under the control of cauliflower mosaic virus (CaMV) 35S and figwort mosaic virus (FMV) (Richins *et al.*, 1987) constitutive promoters (SBPase and FBP aldolase, respectively) and the *A. thaliana* RbcS2 promoter (AGPase). The construct details are given in Supplementary Fig. S1. This recombinant plasmid was introduced into wild-type tobacco (*N. tabacum* cv. Samsun) using *Agrobacterium tumefaciens* strain LBA4404 via leaf disc transformation (Horsch *et al.*, 1985), and the shoots were regenerated on Murashige and Skoog medium containing hygromycin (20 mg l^−1^) and cefotaxime (400 mg l^−1^). Hygromycin-resistant primary transformants (T_0_ generation) with established root systems were transferred to soil and allowed to self-fertilize. Sixty independent lines were generated of which 10 single T-DNA insert lines were taken forward for further analysis in the T_2_ generation. Control plants (CN) used in this study were null segregant plants from the selected transgenic lines, verified by PCR for non-integration of the transgene and absence of overexpression of all three transgenes by qRT-PCR, immunoblots, and proteomics analysis.

### Plant growth conditions and biomass measurements

T_2_ seeds (homozygous and CN) were germinated on soil in controlled-environment chambers at an irradiance of 250 μmol m^−2^ s^−1^, 22 °C air temperature, and relative humidity of 60%, in a 16 h photoperiod. Ten days after sowing, seedlings were transferred to individual 8 cm pots and grown under the same conditions for 13–14 d. At 23–24 d after sowing, plants were transferred into 4 liter pots and moved to a controlled-environment glasshouse (16 h photoperiod, 25 °C–33 °C day/20 °C night, with natural light supplemented under low light induced by cloud cover with high-pressure sodium light bulbs, giving 380–1000 μmol m^−2^ s^−1^ from the pot level to the top of the plant). Plants were distributed in randomized positions in 6–12 blocks and watered regularly with Hoagland’s nutrient medium (Hoagland and Arnon, 1950). Plants were positioned such that at maturity, a near-to-closed canopy was achieved. Plants were harvested for biomass at 25, 46, and 56 d after sowing. Stem length and number of leaves per plant were recorded and the total leaf area per plant was measured with a conveyor-belt scanner (LI-3100C Area Meter; LI-COR, Lincoln, NE, USA). Plants were subsequently separated into leaf and stem fractions and dried to constant weight at 60–70 °C, after which dry weight was determined for each fraction.

A smaller-scale experiment to measure root biomass was conducted in controlled-environment chambers. Irradiance ranged from 250 μmol m^−2^ s^−1^ at pot level to 450 μmol m^−2^ s^−1^ at the top of the canopy the day before measurements were taken. Temperature was kept constant at 23 °C with a relative humidity of 65–70%. Pots were filled with soil and weighed, ensuring a net weight of 145 g at the time seeds were sown. Six plants per line were harvested after 37 d, cut at the stem 1 cm above soil level, and dried at 60–70 °C for 10 d. Weights were recorded for pots containing roots and controls with the same amount of soil but no dried roots.

### Chlorophyll fluorescence imaging

Chlorophyll fluorescence parameters were obtained using a chlorophyll fluorescence imaging system (Technologica, Colchester, UK; Barbagallo *et al.*, 2003; Baker and Rosenqvist, 2004) fitted with a gas-controlled system where the seedlings were exposed to ambient levels of CO_2_ (400–470 ppm), 2% O_2_, and a vapour pressure deficit (VPD) of ∼1 kPa. The operating efficiency of PSII photochemistry, *F*_q_′/*F*_m_′, was calculated from measurements of steady-state fluorescence in the light (*F*′) and maximum fluorescence (*F*_m_′) following a saturating 800 ms pulse of 6300 μmol m^−2^ s^−1^ photosynthetic photon flux density (PPFD), and using the following equation: *F*_q_′/*F*_m_′=(*F*_m_′−*F*′)/*F*_m_′ (Oxborough and Baker, 1997; Baker *et al.*, 2001). Images of *F*_q_′/*F*_m_′ were taken under a stable PPFD of 500 μmol m^−2^ s^−1^ or 800 μmol m^−2^ s^−1^ depending on the experiment. Plants were measured 21–22 d after sowing.

### Leaf gas exchange measurements

Measurements were conducted using a portable infrared gas analyser with an integrated light source (LI-COR 6800; LI-COR, Lincoln, NE, USA). The gas flow rate was kept constant at 500 μmol s^−1^ and the air relative humidity in the leaf chamber was maintained at 55–70% depending on the experiment. Plants were measured within the first 7 h of the photoperiod to minimize diurnal effects on stomatal conductance and photosynthetic activity, apart from diurnal measurements. Young developing leaves were measured as well as mature leaves (positions 8–14 from the bottom of the plant) before the plants began flowering.

#### *A/C*_i_ response measurements

To assess the response of CO_2_ assimilation (*A*) and stomatal conductance (*g*_s_) to intercellular CO_2_ concentration (*C*_i_), leaves were initially acclimated at a saturating irradiance of 1500 μmol m^−2^ s^−1^ and a reference CO_2_ concentration of 400 ppm to mimic ambient levels. Following stabilization, CO_2_ levels were adjusted incrementally (400, 300, 200, 100, 50, 400, 500, 600, 800, 1000, 1200, 1500, and 2000 ppm). Measurements were recorded after *A* and *g*_s_ reached a new steady state (1–2 min between collection of data points) with a leaf temperature range of 26–29 °C. From these response curves, the maximum Rubisco carboxylation rate (*V*_cmax_), maximum electron transport rate (*J*_max_), and triose-phosphate utilization (TPU) were determined using the equations of Farquhar *et al.* (1980) and the R package Plantecophys (Duursma, 2015).

#### PPFD step-response measurements

To assess the response of *A* and *g*_s_ to changes in light intensity, leaves were initially acclimated at a low PPFD of 100 μmol m^−2^ s^−1^ and a reference CO_2_ concentration of 400 ppm until *A* and *g*_s_ reached steady-state levels. After recording *A* and *g*_s_ at these levels, the PPFD was increased in a single step to 1500 μmol m^−2^ s^−1^ and *A* and *g*_s_ were recorded every 20 s for 30 min to capture their response during light induction. The leaf temperature ranged from 24 °C to 27 °C.

#### Diurnal measurements

To assess changes in *A* and *g*_s_ across the photoperiod, *in situ* leaf gas exchange measurements were conducted every 2 h between 07.30 h and 19.30 h. PPFD and temperature levels in the leaf chamber were maintained throughout the measurements to match the environmental conditions in the greenhouse.

### Stomatal density measurements

Stomatal density was measured using leaf surface impressions. Silicone impression material (Xantopren, Heraeus, Germany) was used following the methods described by Weyers and Johansen (1985). Following gas exchange measurements, impressions were taken from the same site on 4–8 leaves per line. Stomatal density was determined using light microscopy (Olympus BX60, Essex, UK) at ×100 magnification. Each impression was analysed using an average of six technical replicates.

### Protein extractions, immunoblotting, and proteomic analysis

Leaf discs were ground in dry ice and protein extractions performed as described in Lopez-Calcagno *et al.* (2017) and using the nucleospin RNA/Protein kit (Macherey-Nagel, http://www.mn-net.com/). Protein quantification was performed using the protein quantification kit from Macherey-Nagel. Samples were loaded on an equal protein basis (5–10 μg per well depending on the experiment), separated using 12% (w/v) SDS–PAGE, transferred to nitrocellulose membranes, and probed using antibodies raised against SBPase (Lefebvre *et al.*, 2005), FBP aldolase (Simkin *et al.*, 2015), AGPase small subunit, and Actin (AS111739 and AS132640, respectively from Agrisera, via Newmarket Scientific, UK). Proteins of interest were detected using horseradish peroxidase conjugated to the secondary antibody and ECL chemiluminescence detection reagent (Amersham, Buckinghamshire, UK). Membranes were also Ponceau stained before blocking to confirm protein transfer and loading.

#### Sample preparation for proteomic analysis and LC-MS/MS analysis

Protein pellets were solubilized in 50 mM ammonium bicarbonate, followed by reduction with DTT (5 mM) and alkylation with iodoacetamide (15 mM). The samples were then digested with Sequencing Grade Modified Trypsin (Promega) for 24 h. Post-digestion, peptide concentrations were estimated using the Pierce Quantitative Fluorometric Peptide Assay. The samples were subsequently dried, resuspended in 0.1% formic acid, and normalized to the same concentration ready for LC-MS/MS analysis.

LC-MS/MS analysis was conducted using a Dionex Ultimate 3000 RSLC nanoUPLC (Thermo Fisher Scientific Inc., Waltham, MA, USA) system coupled with a QExactive Orbitrap mass spectrometer (Thermo Fisher Scientific Inc.). The extracted peptides were initially separated by reverse-phase chromatography at a flow rate of 300 nl min^–1^ on a Thermo Scientific reverse-phase nano Easy-spray column (Thermo Scientific PepMap C18, 2 µm particle size, 100 Åpore size, 75 µm i.d. × 50 cm length). Peptides were loaded onto a pre-column (Thermo Scientific PepMap 100 C18, 5 µm particle size, 100 Å pore size, 300 µm i.d. × 5 mm length) from the Ultimate 3000 autosampler with 0.1% formic acid for 3 min at a flow rate of 15 µl min^−1^. After this period, the column valve was switched to allow elution of peptides from the pre-column onto the analytical column. Solvent A comprised 0.1% formic acid in water, and solvent B consisted of 80% acetonitrile, 20% water, and 0.1% formic acid. The linear gradient employed was 2–40% B in 90 min, with a total run time of 120 min including column washing and re-equilibration.

The eluted peptides were sprayed into the mass spectrometer using an Easy-Spray source (Thermo Fisher Scientific Inc.). All *m/z* values of eluting ions were measured in an Orbitrap mass analyzer, set at a resolution of 35 000, scanning between *m/z* 380 and 1500. Data-dependent scans (Top 20) were employed to automatically isolate and generate fragment ions by higher energy collisional dissociation (HCD, NCE: 25%) in the HCD collision cell, with the resulting fragment ions measured in the Orbitrap analyzer at a resolution of 17 500. Singly charged ions and ions with unassigned charge states were excluded from MS/MS selection, and a dynamic exclusion window of 20 s was employed.

All MS/MS samples were analysed using Mascot (Matrix Science, London, UK; version Mascot in Proteome Discoverer 2.4.1.15). Mascot was set up to search against the *N. tabacum* proteome (Uniprot, downloaded on 2 June 2021) and a common contaminant database, assuming trypsin digestion. Mascot searches were performed with a fragment ion mass tolerance of 0.100 Da and a parent ion tolerance of 20 ppm. Carbamidomethylation of cysteine was specified as a fixed modification, while deamidation of asparagine and glutamine and oxidation of methionine were specified as variable modifications. Peak areas of identified peptides were calculated for label-free quantification.

Scaffold (v4.10.0, Proteome Software Inc., Portland, OR, USA) was used to validate MS/MS-based peptide and protein identifications. Peptide identifications were accepted if they could be established at >95.0% probability by the Scaffold Local false discovery rate (FDR) algorithm. Protein identifications were accepted if they could be established at >99.0% probability and contained at least two identified peptides. Protein probabilities were assigned by the Protein Prophet algorithm (Nesvizhskii *et al.*, 2003). Proteins containing similar peptides and which could not be differentiated based on MS/MS analysis alone were grouped to satisfy the principles of parsimony. Proteins sharing significant peptide evidence were grouped into clusters.

### RNA extraction and transcriptomics

Leaf discs of the relevant transgenic and azygous lines were ground in dry ice, and RNA extractions were performed following the nucleospin RNA/Protein kit instructions (Macherey-Nagel). RNA was used for target gene analysis in T_1_ plants using qPCR analysis. One or two homozygous and azygous plants were sampled for each line, and expression of each transgene was assessed as folds of the level of expression of two housekeeping genes; PP2A (X97913) and EF (LOC107826390). Three technical replicates were used for each gene assessed. In T_2_ plants, RNA was used for RNA-seq which was performed by Novogene. After checking RNA read quality using FastQC (https://www.bioinformatics.babraham.ac.uk/projects/fastqc/), a classification-based quantification was performed using kallisto (Bray *et al.*, 2016). In short, a kallisto index was built with the reference transcriptome of *N. tabacum* (Edwards *et al.*, 2017) appending the sequences of the introduced genes. Kallisto quant was used to quantify abundance of paired-end reads with default parameters. The resulting abundance files were used to generate a count table in R version 4.0.3 (R Core Team, 2020) using package tximport (Soneson *et al.*, 2015). Genes with low counts (<10 reads) were removed, and differential expression analysis was performed in R using DESeq2 (Love *et al.*, 2014). Genes with an adjusted *P*-value <0.001 and a fold2logchange ratio >1.5 (or < −1.5) were highlighted as differentially expressed and included in Supplementary Table S1.

### Soluble sugar and starch determination

Samples from the fully expanded leaves used for *A/C*_i_ response curves were collected at mid-day and flash frozen. Ethanolic extracts from 20 mg of frozen plant material were used to determine sucrose, glucose, and fructose as in Cross *et al.* (2006). For starch determination, pellets from the ethanolic extract preparations were solubilized by heating them to 80 °C in 0.1 M NaOH for 40 min. After neutralizing the pH with an HCl/sodium acetate solution, the suspension was digested overnight with amyloglucosidase and amylase. The glucose content of the supernatant was then used to determine the starch content in the sample. Protein was quantified from these samples and carbohydrates were normalized to this.

### Statistical analysis

All statistical analyses were performed using R (https://www.r-project.org/, version 4.0.5). ANOVA considering block effect when relevant, followed by post-hoc *t*-tests or Tukey tests were carried out.

## Results

### Production and selection of tobacco transformants

**Fig. 1.**
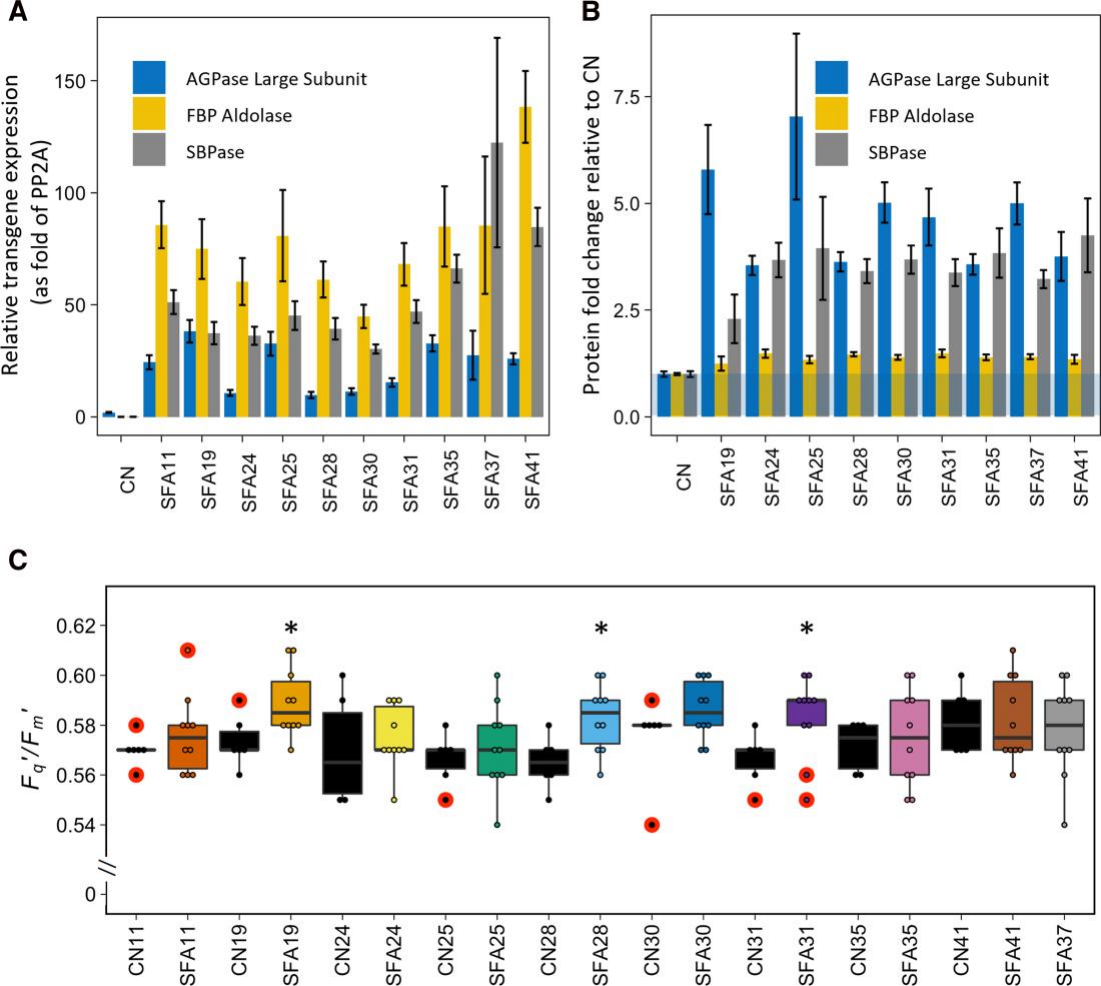
Production and selection of triple overexpressor lines. (A) qPCR/transcript abundance in T_1_ plants. (B) Protein quantification; relative spectral counts for the target proteins in fully expanded leaves of T_2_ plants. (C) *F*_q_′/*F*_m_′ at low O_2_ and 500 μmol m^−2^ s^−1^ of T_2_ plants 21–22 d after sowing. *n=*6–10. *P<0.05 (linear mixed model analysis accounting for block as random effect was carried out; comparisons were done only between each SFA and corresponding CN). In boxplots: the box represents the middle 50% of the data (first to thirrd quartile, Q1–Q3); the mid-line indicates the median (Q2); whiskers extend to the most extreme data points within 1.5× the interquartile range (IQR); and outliers beyond this range are shown as large red dots.

To explore the impact of simultaneously overexpressing SBPase, FBP aldolase, and AGPase, an overexpression construct for these three genes (Supplementary Fig. S1) was used for tobacco transformation. Over 60 independent T_0_ lines were generated, of which 37 had a single T-DNA insert. Ten single T-DNA insert lines were selected at the T_0_ stage based on detectable expression of all three transgenes, and T_1_ seed were grown to generate homozygous lines. Nine homozygous lines alongside eight azygous segregants (which have lost the T-DNA of interest and from this point forward were used as control plants) were successfully identified and further characterized. Transgene expression analysis by qPCR in T_1_ plants confirmed expression of all three transgenes in the nine selected lines, with no expression in selected control lines (Fig. 1A; Supplementary Fig. S2). Immunoblot analysis further confirmed the identity of these lines, with increased levels of the three target proteins in the homozygous lines and no changes in the control lines (Supplementary Fig. S3). Lines were further characterized in the T_2_ generation.

Proteome analysis using tandem MS coupled with LC (LC-MS/MS) confirmed the consistent accumulation of the three target proteins in T_2_ homozygous transgenic lines, with no other consistent changes in levels for any other protein. Across the selected lines, the abundance of SBPase increased between 2.3- and 4.2-fold of CN levels, FBP aldolase 1.2- to 1.5-fold, and AGPase large subunit to 3.5- to 7-fold compared with the controls (CN) (Fig. 1B). It also confirmed that the levels of both AGPase subunits were elevated, suggesting that this approach led to an increase of the functional form of this protein. However, the increase in the AGPase small subunit did not match that of the overexpressed large subunit, only reaching ∼2-fold of CN (Supplementary Fig. S4). In addition to this, an RNA-seq analysis was carried out in a subset of the lines (SFA19, SFA28, and SFA30), confirming that only the three target genes (SBPase, FBP aldolase, and AGPase) were consistently changed in the lines produced (Supplementary Table S1).

### Photosynthetic characterization: chlorophyll fluorescence, *A*/*C*_i_ response curves, PPFD step response curves, and diurnal CO_2_ assimilation

To test if the changes in enzyme levels had a positive effect on photosynthesis, chlorophyll fluorescence imaging was used as a quick screen on young plants (21–22 d after sowing) grown under control conditions (22 °C, 250 μmol m^−2^ s^−1^ and a 16 h photoperiod). This analysis was done at low O_2_ and an irradiance of 500 μmol m^−2^ s^−1^, and it showed larger average operating efficiency of PSII photochemistry (*F*_q_′*/F*_m_′) for the transgenics than the grouped controls (CN), with significantly increased *F*_q_′*/F*_m_′ in three of the nine lines (Fig. 1C).

**Fig. 2.**
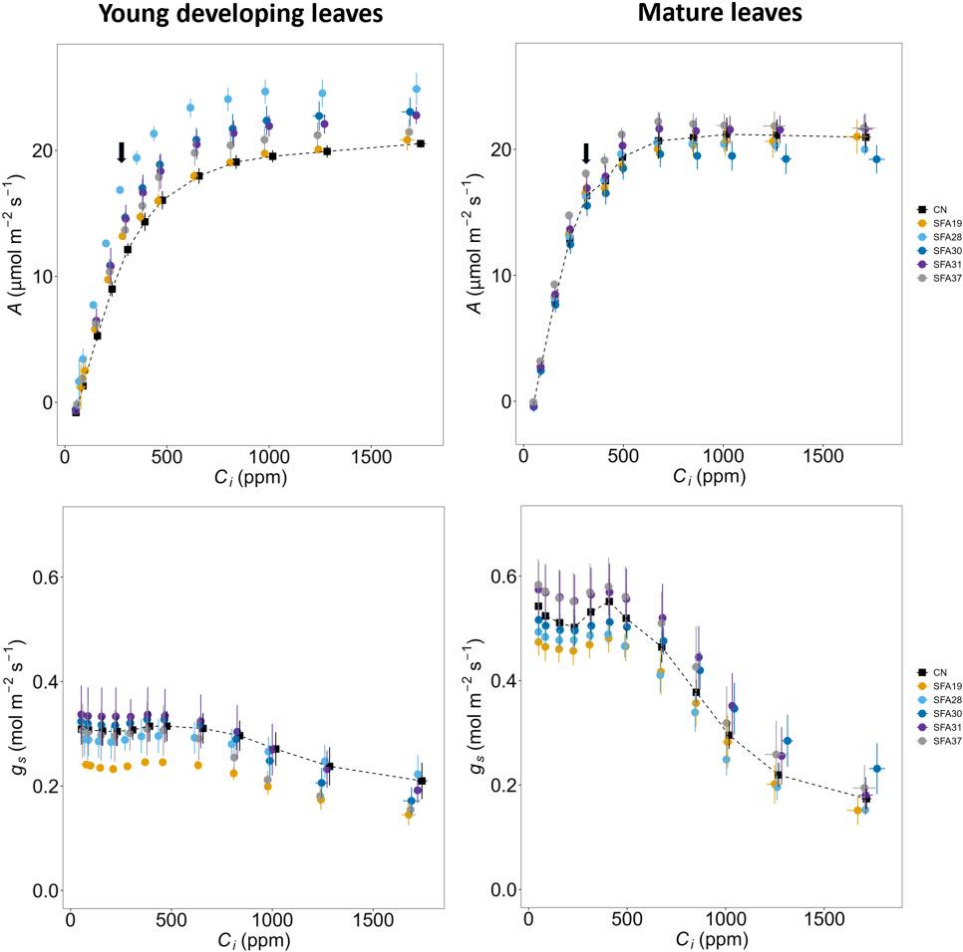
CO_2_ assimilation and stomatal conductance as a function of increasing intercellular CO_2_ concentrations in young developing leaves and mature leaves. CO_2_ assimilation (*A*) and stomatal conductance (*g*_s_) as a function of increasing intercellular CO_2_ concentrations (*C*_i_) in young developing leaves (left) and mature leaves (right) of transgenic (SFA) and control plants (CN) grown in the glasshouse. Measurements were made at an irradiance of 1500 µmol m^−2^ s^−1^ with a range in leaf temperatures of 26–28 °C. Black arrows indicate *C*_i_ levels corresponding to ambient CO_2_ levels. *n=*3–5.

To further test the impact of increased levels of SBPase, FBP aldolase, and AGPase abundance in carbon fixation, transgenic and control plants were grown in greenhouse conditions (16 h photoperiod, 25–33 °C day/20 °C night, with natural light supplemented under low light induced by cloud cover with high-pressure sodium light bulbs) and photosynthesis was determined using gas exchange on a young developing leaf and a mature leaf of each plant. The data show that in the young developing leaves, the rate of CO_2_ assimilation (*A*) at different intercellular CO_2_ partial pressures (*C*_i_) and saturating irradiance was increased compared with CN in six out of the nine lines measured (Fig. 2; Supplementary Fig. S5A). However, in mature leaves, no consistent increase in *A* or change in *g*_s_ was evident (Fig. 2; Supplementary Fig. S5B). Analysis of the photosynthetic parameters *V*_cmax_, *J*_max_, and TPU on five selected lines in young developing leaves showed a significantly higher *V*_cmax_ in line SFA28 versus CN, and higher average values for all three parameters in four out of the five lines analysed versus CN (Table 1). In contrast, in mature leaves, although some lines displayed higher average values than CN, there was no consistent trend and no significant differences for any of the lines in the three parameters analysed (Table 1). This was repeated in two additional experiments with similar results.

**Table 1.** Maximum Rubisco carboxylation rate (*V*_cmax_), maximum electron transport rate (*J*_max_), and triose-phosphate utilization (TPU) in young expanding and mature leaves of control (CN) and SFA lines

Genotype	Parameters (µmol m^−2^ s^−1^)					
	Young expanding leaves			Mature leaves		
	*V*_cmax_	*J*_max_	TPU	*V*_cmax_	*J*_max_	TPU
CN	47.5±2.9	93.8±3.2	7.4±0.1	62.1±4.8	101.8±7.8	7.4±0.4
SFA19	54.1±1.6	91.5±0.9	NI	66.2±2.3	94.7±0.9	6.7±0.1
SFA28	68.6±2.7*	108.5±8.1	7.9±0.6	62.5±1.5	98.7±1.6	6.9±0.2
SFA30	59.7±4.3	108.0±6.6	8.1±0.5	60.8±2.7	96.6±4.5	6.7±0.4
SFA31	59.1±7.3	106.1±4.2	8.1±0.01	66.0±5.0	106.0±7.7	7.4±0.4
SFA37	54.2±4.3	98.9±5.6	7.4±0.3	70.9±2.4	109.3±2.6	7.2±0.1
N	3–5	2–4	2–4	3–5	3–5	3–4

These parameters are reported at 25 °C and were derived from the *A*/*C*_i_ response curves from Fig. 2. Statistical differences obtained from one-way ANOVAs between SFA lines and the control plants are shown in bold (* *P*<0.05). Average ±SE values and number of replicates after removing outliers are shown. NI, not included

To further explore whether mature leaves in the transgenic lines might have changes in *A* during non-steady-state conditions, lines SFA19, SFA28, SFA30, SFA31, and SFA37 were subjected to an increase in PPFD. No significant changes in the speed or amplitude of *A* or *g*_s_ induction were found when leaves were exposed to low to high light (Supplementary Fig. S6). Additionally, we looked at the integrated diurnal CO_2_ assimilation in mature leaves and assimilation at midday, which also revealed no significant differences (Supplementary Fig. S7). While assessing photosynthesis, stomatal impressions were collected to evaluate stomatal density; again no significant differences were found (Supplementary Fig. S8).

### Carbohydrate measurements

**Fig. 3.**
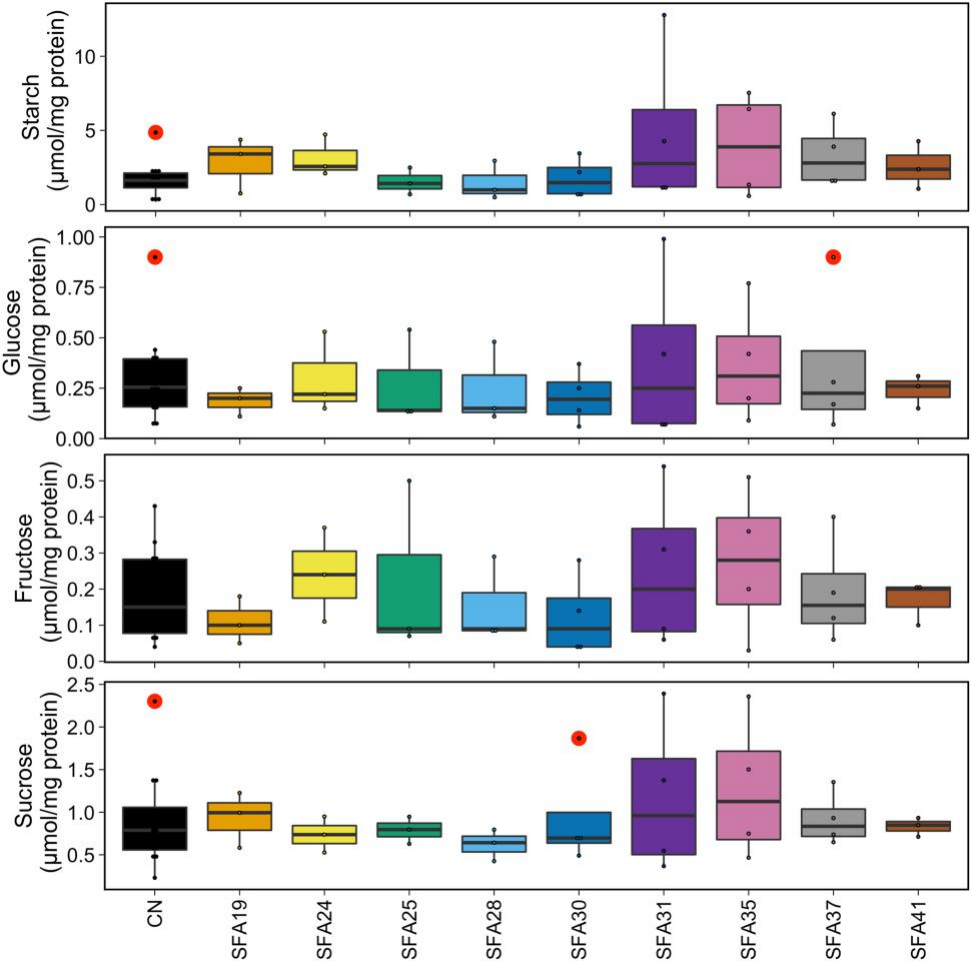
Overexpression of SBPase, FBP aldolase, and AGPase does not consistently change the carbohydrate profile at midday. *n*=3–4 for transgenics, *n*=10–13 for CN. No statistically significant changes were found in any of the four carbohydrates measured (linear mixed models -lmer- accounting for block as random effect). In boxplots: the box represents the middle 50% of the data (first to third quartile, Q1–Q3); the mid-line indicates the median (Q2); whiskers extend to the most extreme data points within 1.5× the interquartile range (IQR); and outliers beyond this range are shown as red dots.

AGPase catalyses the first committed step of starch biosynthesis in higher plants, hence we hypothesized that increasing AGPase capacity by increasing the levels of this enzyme would allow any additional carbon fixed to be moved into starch by the removal of the bottlenecks represented by SBPase and FBP aldolase or negative feedback on photosynthesis by Pi accumulation. Consequently, leaf carbohydrate measurements, including starch quantification, were done on the mature leaves used for gas exchange (Fig. 3). No consistent significant changes were detected in either starch or the soluble sugars measured (sucrose, fructose, and glucose).

### Biomass yield determination

**Fig. 4.**
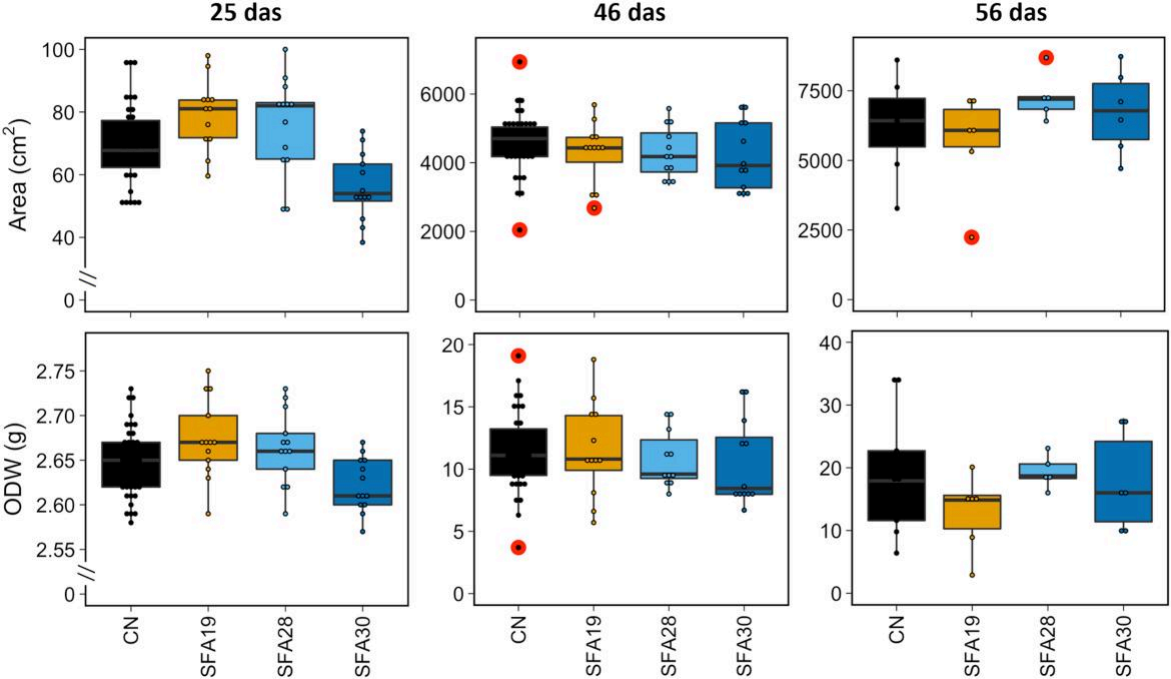
Simultaneous increases in SBPase, FBP aldolase, and AGPase do not affect tobacco biomass in glasshouse-grown plants at 25 d (*n*=13), 46 d (*n*=12), and 56 d after sowing (*n*=6 for each SFA line, *n*=9 for CN). Leaf area (top) and total above-ground dry weight (bottom) (ODW). ANOVA considering block effect was used to compare control groups and transgenics.The CN group corresponds to pooled azygotes from each line which were not significantly different from each other; statistical analysis compared each overexpressing line with its corresponding CN at 25 d and 46 d after sowing. At the 56 d after sowing time point, the CN were not separated by line and hence the comparison was made against the pooled CN group. In boxplots: the box represents the middle 50% of the data (first to third quartile, Q1–Q3); the mid-line indicates the median (Q2); whiskers extend to the most extreme data points within 1.5× the interquartile range (IQR); and outliers beyond this range are shown as larger red dots.

Given that significant increases in *F*_q_′*/F*_m_′ were observed in young plants at light intensities of 500 μmol m^−2^ s^−1^, and consistent increases in *A/C*_i_ response curves were only found in young expanding leaves, no consistent differences in photosynthesis were found later in mature leaves. We next assessed total leaf area and above-ground biomass at three different times in development—25, 46, and 56 d after sowing—for a subset of the transgenic lines (SFA19, SFA28, and SFA30) (Fig. 4). The biomass of the full set of lines was also measured at flowering onset, ∼56 d after sowing (Supplementary Fig. S9). No impact on total leaf area or above-ground biomass was found at any of the time points for any line. Additionally, to examine whether the early increases in photosynthesis might have influenced carbon allocation to the root system, root biomass was measured 37 d after sowing. No consistent significant differences between the transgenics and CN plants were found (Supplementary Fig. S10).

## Discussion

Previously it has been shown that increases in SBPase and FBP aldolase activity either separately or together in tobacco resulted in increased photosynthesis and biomass when plants were grown in greenhouse conditions (Simkin *et al.*, 2015). Overexpression of AGPase in leaves has also shown that this manipulation has the possibility to increase photosynthesis and yield in rice (Oiestad *et al.*, 2016). A model developed to assess the limitations on photosynthesis under fixed nitrogen conditions identified >20 proteins which would need adjustment in concentrations to optimize photosynthetic carbon metabolism; of these, two enzymes in the CBB cycle, SBPase and FBP aldolase, and the starch biosynthetic enzyme AGPase were the targets with the largest increase (Zhu *et al.*, 2007).

The aim of the work in this study was to explore a minimal hypothesis raised by this model that combining AGPase overexpression together with that of SBPase and FBP aldolase could further improve photosynthesis and plant biomass in tobacco and hence increase productivity. Homozygous transgenic tobacco lines were produced and grown in greenhouse conditions and a range of photosynthetic and growth parameters were determined.

In young plants (21–22 d post-planting), chlorophyll fluorescence image analysis revealed increases in PSII operating efficiency under low O_2_, suggesting an increase in CO_2_ assimilation capacity in these transgenic plants. This was supported by *A/C*_i_ analysis of young leaves of the transgenic lines that revealed a clear trend of increased carbon assimilation in most lines. However, a similar analysis of mature leaves on the same plants did not reveal a consistent increase in *A* in the transgenics. Although in these older leaves there may be some TPU limitation above 600 ppm hindering any increases in CO_2_ assimilation, we also did not see any significant changes at lower CO_2_ concentrations (Yin *et al.*, 2021). In keeping with these results, no changes were observed in the above-ground biomass in any of the lines analysed at any of the times analysed, nor were there consistent changes in root growth in young plants. Below we discuss these results and possible explanations for the phenotype found.

In the transgenic T_2_ homozygous lines, SBPase levels were increased to 2.3- to 4.2-fold of CN, FBP aldolase to 1.2- to 1.5-fold, and AGPase large subunit to 3.5- to 7-fold (Fig. 1B). It was also confirmed that the levels of the small AGPase subunit were elevated (Supplementary Fig. S4), suggesting that this approach led to an increase of the holoenzyme form of this protein. The achieved protein levels in our target enzymes are within the range suggested by the outputs of the model of Zhu *et al.* (2007), albeit not exactly as predicted. However, the authors of the model state that it is unlikely that the model predictions are totally accurate given that the model was parameterized using data from a range of species (Zhu *et al.*, 2007). To improve on this model*, in silico* sensitivity analysis could be used where all parameters are varied stochastically to identify the variation in the control exhibited by the target enzyme. Alternatively, the model could be parameterized using enzyme kinetic data obtained specifically from the target species (*N. tabacum*). Another limitation with this type of model is that it includes only a subset of metabolic reactions and does not account for organism-level complexities such as multi-compartments and multi-tissues, nor does it consider the changes occurring throughout the plant life cycle.

Even if the disparity in enzyme levels achieved between our plants and those suggested by the model was the reason for the lack of uplift in photosynthesis, it would be a significant challenge to engineer plants with the exact changes as suggested by the model and would involve screening of a large number of transformants with no guarantee of success. Additionally, it may not be possible to increase the levels of these enzymes further without having a detrimental impact on the plant through diversion of nitrogen from essential roles to support the additional protein required as the model was based on no increase in protein nitrogen. To achieve this balance in nitrogen investment, the model included down-regulation of the glycine decarboxylase (GDC) complex of the photorespiratory pathway, among other targets, to balance the increased level of other enzymes. However, due to the well-known negative effect on growth of significant reductions in, for example, proteins of the GDC complex (Engel *et al.*, 2007; Zhou *et al.*, 2013; Lin *et al.*, 2016), this approach would necessitate very tight control over the amount of protein removed, which further complicates the generation of these multitarget transgenics. Given the difficulties of finely controlling the level of more than three genes, we avoided tackling these reductions at this stage. However, this is something that should be considered in future studies.

Metabolic control analysis (MCA) is an empirical approach to quantify the control of flux an individual enzyme exerts on a pathway, termed the flux control coefficient (FCC) (Kacser and Burns, 1973; Heinrich and Rapoport, 1974; Fell, 1997). MCA allows for all enzymes in a pathway to share control of flux, albeit not equally, and has been used to identify enzymes in the CBB cycle controlling CO_2_ assimilation. This work also showed that the FCC for an individual enzyme is not constant and changes with environmental conditions and developmental stage of the plant. Taking this approach it was shown that, in addition to Rubisco, SBPase, FBP aldolase, and tranketolase were potential targets for improving photosynthesis (Stitt and Schulze, 1994; Raines, 2003). Although overexpression of SBPase and FBPaldolase has had positive impacts on photosynthesis (Simkin *et al.*, 2015, 2017), this has not been the case for transketolase, where overexpression resulted in smaller plants and lower photosynthesis due to a negative and unexpected effect on thiamine biosynthesis (Khozaei *et al.*, 2015). Our results may be explained by a more recent dynamic systems model of photosynthetic carbon metabolism, which proposes that the CBB cycle is organized into interacting subcycles and that some enzymes display a biphasic response to increasing activity (Zhao *et al.*, 2021). According to this model, excessive increases in the activities of enzymes in one subcycle consuming a shared metabolite could cause low concentrations of metabolites in the other subcycles, resulting in reductions in reaction rates and lowering overall CBB cycle flux. It is therefore possible, similar to the transketolase-overexpressing plants, that we have created an imbalance of enzyme activities now in the subcycles of the CBB cycle, particularly given that, like transketolase, FBP aldolase has a biphasic response (Zhao *et al.*, 2021). What is somewhat puzzling is that overexpression of SBPase and FBP aldolase together has been shown to result in significant increases in both photosynthesis and biomass in tobacco grown in the greenhouse (Simkin *et al.*, 2015). Similarly, overexpression of AGPase in rice has also led to increases in productivity (Oiestad *et al.*, 2016). However, effectively, we have an uplift in the small subunit of only 2-fold, and empirical studies in Arabidopsis and rice found that ∼2-fold increases did not enhance photosynthesis under ambient CO_2_ (Obana *et al.*, 2006; Gibson *et al.*, 2011). This suggests that the combination of SBPase and FBPA overexpression with AGPase overexpression has produced an unfavourable imbalance within the system. This could arise either through disruption of CBB cycle metabolite partitioning via fructose-1,6-bisphosphatase, impaired stoichiometry of AGPase subunits affecting holoenzyme activity, or an excess of FBP aldolase relative to the conditions reported by Simkin *et al*. (2015). Resolving these possibilities will require further analysis of *in vivo* enzyme activities and metabolic fluxes.

An important consideration is that the Zhu *et al.* (2007) model was designed to ‘partition optimally with respect to maximizing light-saturated photosynthetic rate for a typical C3 leaf’. In real canopies, however, most leaves are not continuously light saturated throughout their life span. Under our conditions—especially after transfer to the greenhouse—it is likely that only the young, expanding leaves at the top of the canopy (where increases in *A* were detected in the *A/C*_i_ curves) experienced sustained high light, whereas mature leaves (where increases in *A* were not detected in the *A/C*_i_ curves or dynamic measurements; Fig. 2; Supplementary Figs S4, S5) were exposed to subsaturating light most of the time. This suggests that additional factors could also be responsible for the lack of the expected phenotype in mature leaves, and the consequent increase in biomass.

One such factor could be acclimation of lower canopy leaves to low light. If light-harvesting complexes and electron transport chains in these leaves are tuned to subsaturating light conditions, they may be unable to provide sufficient energy to drive the ‘optimized’ CBB cycle during brief periods of saturating light, even when key enzymes are overexpressed. Such acclimation could negate potential benefits and make the extra investment of energy, carbon, and nitrogen in these enzymes counterproductive. Similar limitations of biochemical enhancements under fluctuating light have been noted by Vialet-Chabrand *et al.* (2017).

One last point to consider is that these manipulations may work differently across species. In Arabidopsis and rice, leaf starch acts as a short-term carbohydrate reservoir—often called transitory starch—providing a continued supply of carbohydrate when photosynthesis is not occurring (Sebastian and Samuel, 2012; Rashid *et al.*, 2020). Increasing this pool by enhancing AGPase activity in these species has been shown to boost plant growth (Gibson *et al.*, 2011; Oiestad *et al.*, 2016). In contrast, tobacco is a strong starch accumulator, with reserves that are not fully consumed at night and, in some cultivars, may also be sink limited (Matt *et al.*, 1998; Chu *et al.*, 2022). Since the models considered here (Zhu *et al.*, 2007; Zhou *et al.*, 2013) were based on general C_3_ photosynthesis dynamics rather than a specific species, these may overlook species-specific aspects of metabolism. While our results suggest that the strategy tested here may not work in starch-accumulating species such as tobacco, this strategy could still be effective in other non-starch-accumulating species. Supporting this, a recent modelling study applying the e-photosynthesis model to *Solanum tuberosum* (potato) highlights the need for increases in the same target enzymes described here, among others (Vijayakumar *et al.*, 2024). Further empirical studies in species with larger sinks, such as potato, will be needed to explore this further.

Although these results have shown that this manipulation under our growth conditions was not successful at increasing productivity in *N. tabacum*, there are limitations to the current study, such as the low levels of light, and lack of *in vivo* activity measurements and carbon flux studies, which could have better explained the results presented. However, our results shed light on important considerations should this or similar approaches be taken in future to increase photosynthetic carbon assimilation and crop yields; before disregarding this approach for increasing productivity, consideration should be given to growing these plants under different light and nitrogen regimes, as well as testing the approach in other species.

## Supplementary Material

erag121_Supplementary_Data

## Data Availability

The primary data supporting this study were not made publicly available at the time of publication, with the exception of the RNA-seq dataset, which has been deposited in the NCBI BioProject database under accession number PRJNA1211028. The remaining data that support the findings of this study are available from the corresponding authors upon request.

## Supplementary data

The following supplementary data are available at *JXB* online.

Fig. S1. Construct used for overexpression of SBPase, FBP aldolase, and AGPase.

Fig. S2. Production and selection of triple overexpressor lines.

Fig. S3. Immunoblot analysis of leaf protein extracts from T_1_ generation transgenics.

Fig. S4. AGPase protein quantification.

Fig. S5. CO_2_ assimilation as a function of increasing intercellular CO_2_ concentrations (all lines).

Fig. S6. CO_2_ assimilation and stomatal conductance following a step increase in PPFD.

Fig. S7. Combined accumulated assimilation of CO_2_.

Fig. S8. Overexpression of SBPase, FBP aldolase, and AGPase does not lead to significant differences in stomatal density.

Fig. S9. Overexpression of SBPase, FBP aldolase, and AGPase does not affect biomass in glasshouse-grown plants at the onset of flowering (all lines).

Fig. S10. Overexpression of SBPase, FBP aldolase, and AGPase does not lead to consistent changes in root biomass.

Table S1. RNA-seq analysis confirming that only the three target genes were consistently overexpressed in three of the transgenic lines.

## Abbreviations

*A*: rate of CO_2_ assimilation
AGPase: ADP-glucose pyrophosphorylase
CBB: Calvin–Benson–Bassham
*C*_i_: intercellular CO_2_ partial pressures
CN: control plants
FBP aldolase: fructose-1,6-bisphosphate aldolase
*F*_q_′/*F*_m_′: operating efficiency of PSII photochemistry
*g*_s_: stomatal conductance
PPFD: photosynthetic photon flux density
SBPase: sedoheptulose-1,7-bisphosphatase
