# Supplementary material for: Simultaneous overexpression of three enzymes of chloroplast metabolism fails to improve CO2 assimilation or biomass
Source: J Exp Bot. 2026 Mar 7;77(12):3837–48. doi: 10.1093/jxb/erag121 (PMC13293071; doi:10.1093/jxb/erag121)
Supplement: erag121_Supplementary_Data [file erag121_supplementary_data.pdf]

Supplemental Table S1.- RNAseq analysis confirming that only the three target genes were consistently overexpressed in three of the transgenic lines. Differential expression was tested with Wald test (DESeq2). padj are p-value adjusted by the Benjamini-Hochberg procedure.

description	baseMean	log2FoldChange	p_value	padj
AtAGPase	50,325.99	16.23	0.00	< .001***
AtSBPase	84,175.61	14.19	0.00	< .001***
SlFBPAldolase	54,334.79	14.51	0.00	< .001***

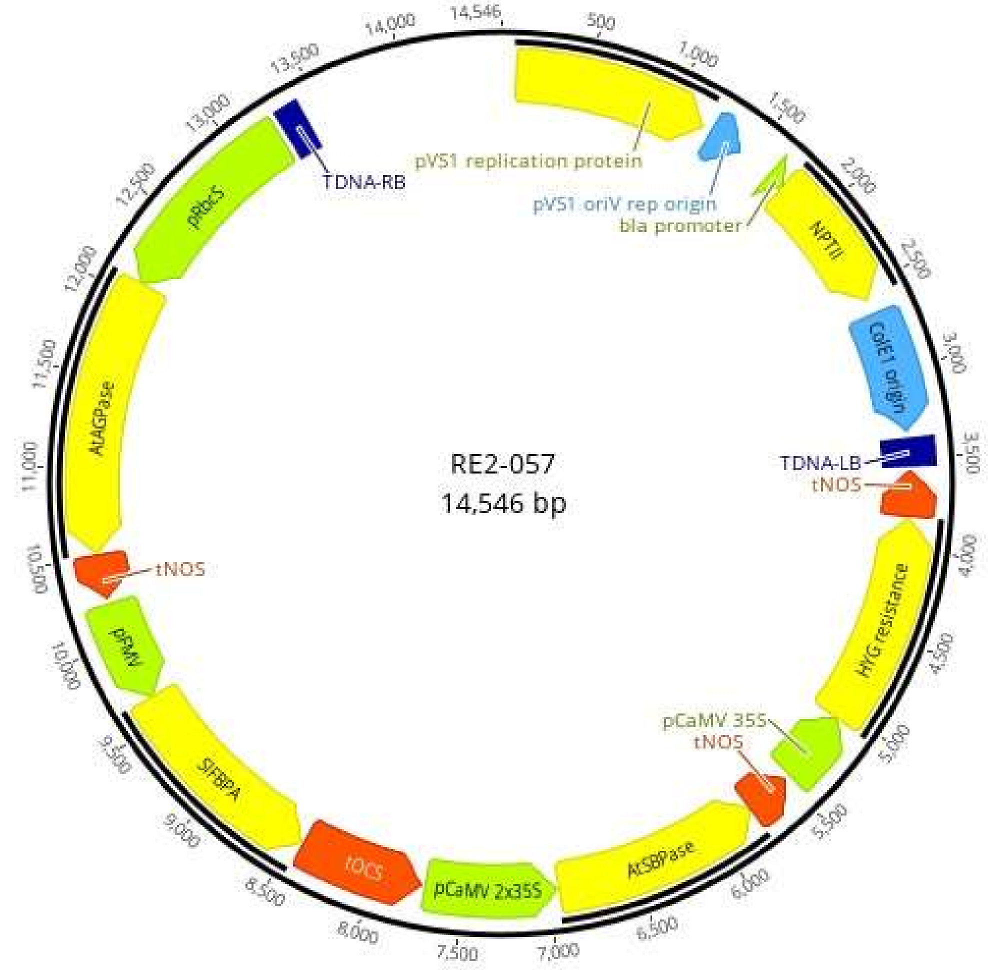

Supplemental Figure S1. Construct used for overexpression of SBPase, FBP Aldolase and AGPase in tobacco.

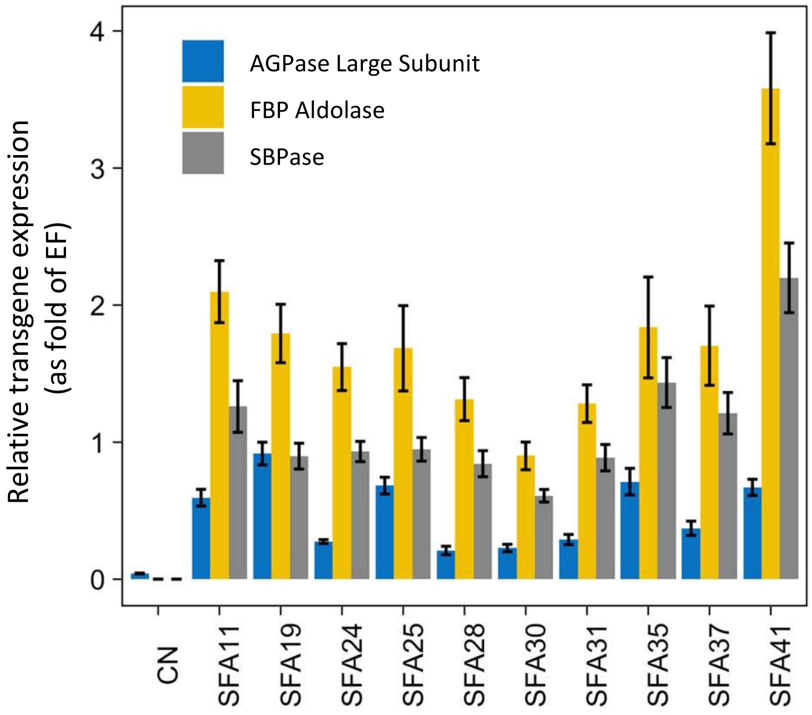

Supplemental Figure S2. Production and selection of triple overexpressor. qPCR/transcript abundance in T1 plants normalized to housekeeping gene EF.

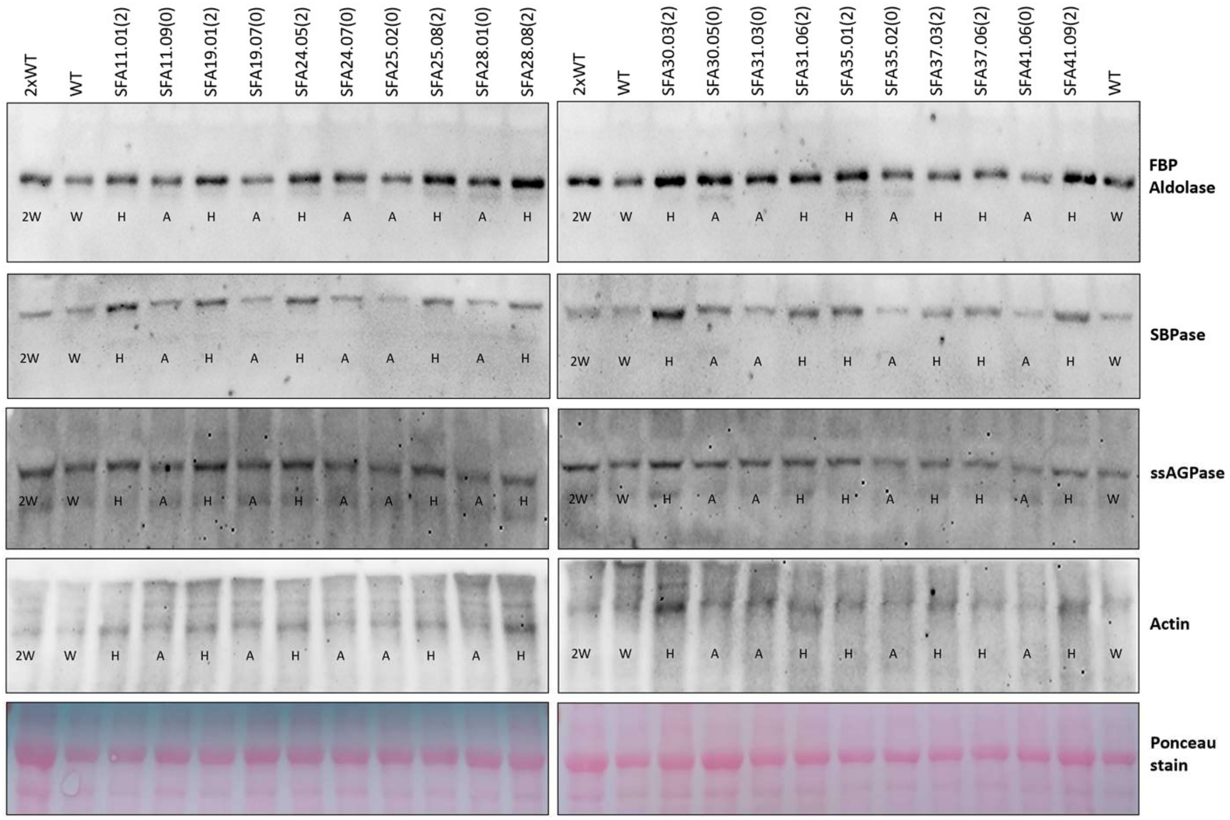

Supplemental Figure S3. Immunoblot analysis of leaf protein extracts from T1 generation. All lines screened using antibodies against SBPase, FBP Aldolase and AGPase small subunit. Actin antibodies and Ponceau Stain were used as loading controls. Insert number included next to each plant label, as well as segregation state on the blots. Homozygous (H), azygous (A) and WT plants included as available.

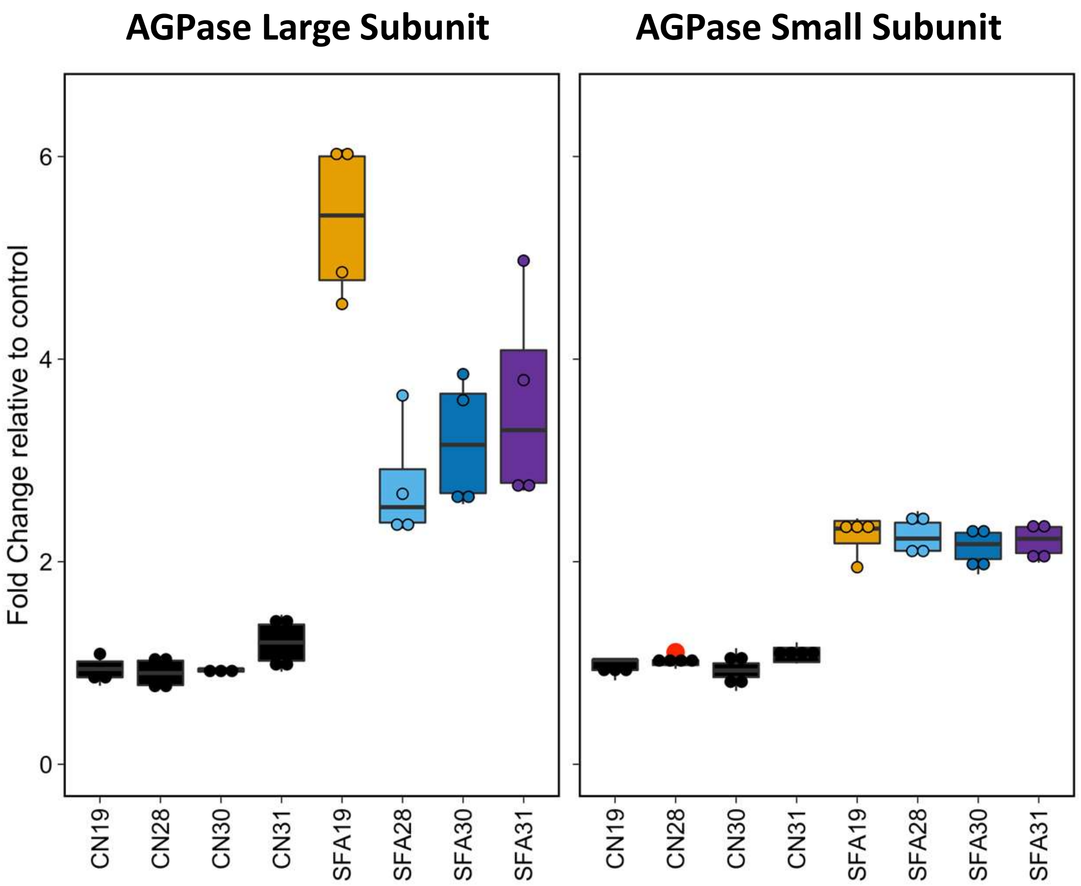

Supplemental Figure S4. AGPase Protein quantification. Relative spectral counts for the two AGPase subunits in mature leaves.

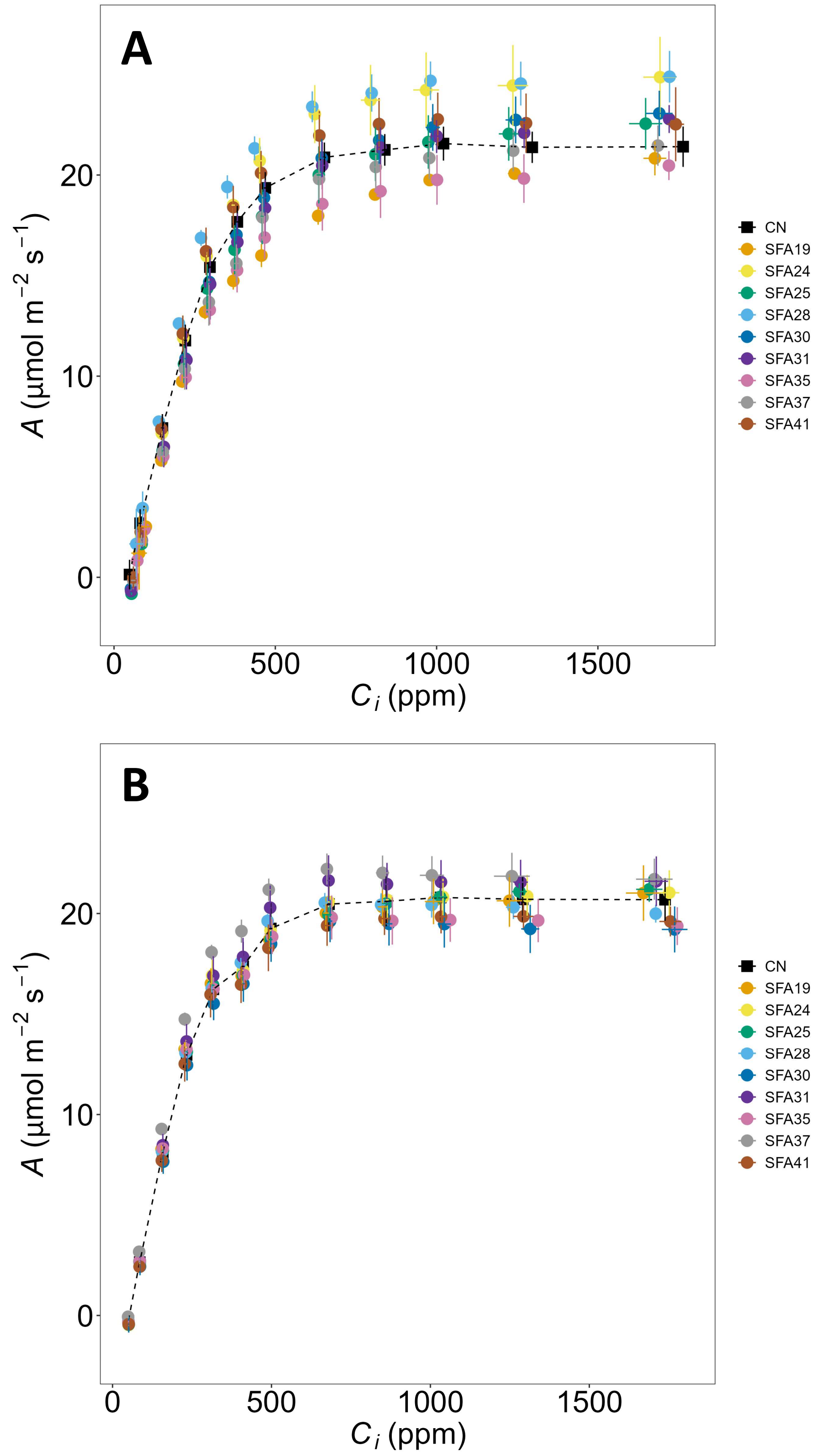

Supplemental Figure S5. CO_2 assimilation (*A*) as a function of increasing intercellular CO_2 concentrations (C_i) in **A)** young developing leaves and **B)** mature leaves of 9 of the transgenic (SFA) and control plants (CN) grown in the glasshouse. Measurements were made at an irradiance of 1500 µmol m^{-2} s^{-1} with a range in leaf temperatures of 26-29 °C. N=3-5.

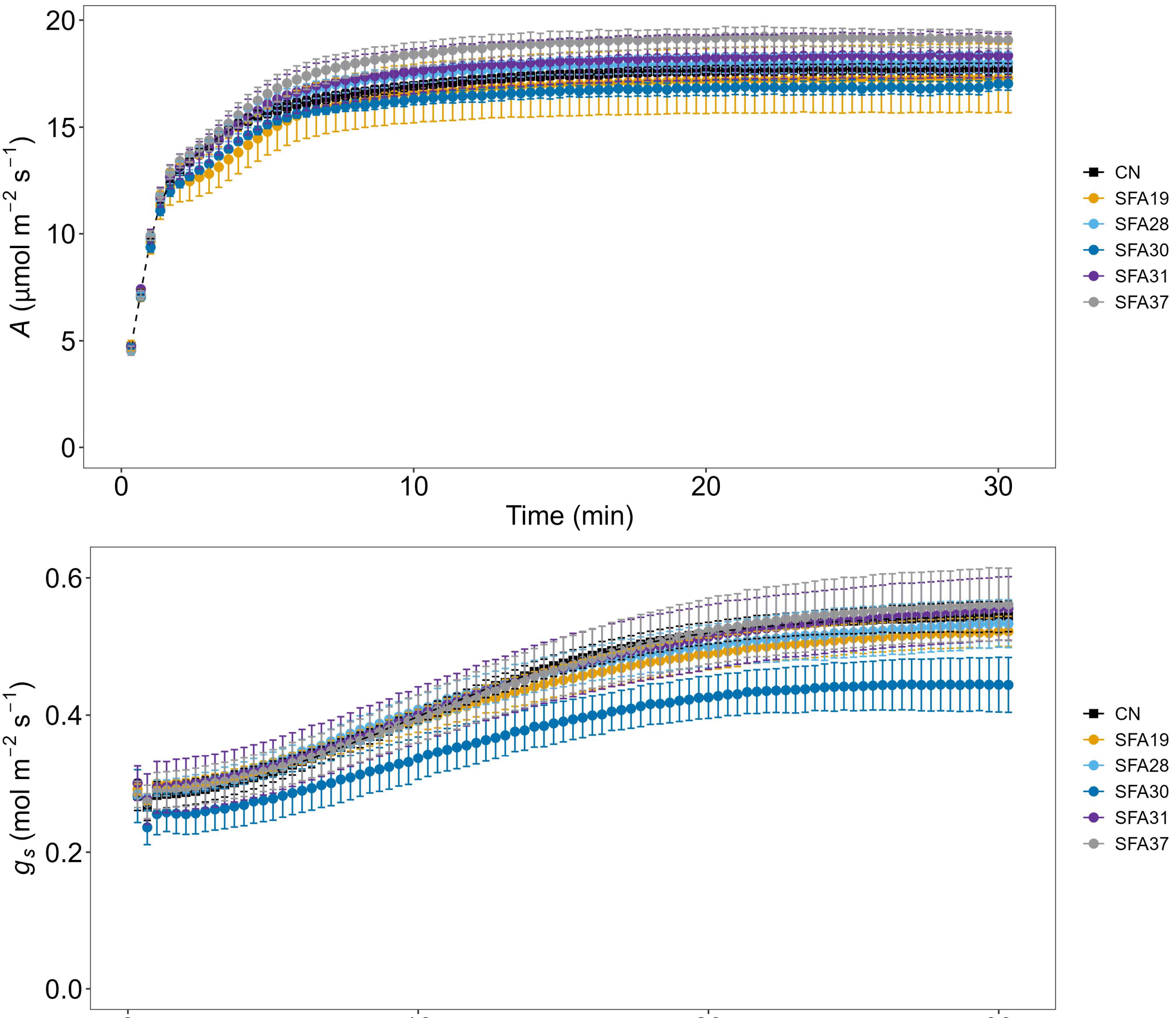

Supplemental Figure S6. CO_2 assimilation (*A*) and stomatal conductance (g_s) following a step increase in PPFD (Photosynthetic Photon Flux Density) from 100 to 1500 µmol m^{-2} s^{-1} in mature leaves of transgenic (SFA) and control plants (CN) grown in the glasshouse. Measurements were made with a range in leaf temperatures of 24-27 °C. N=3-4

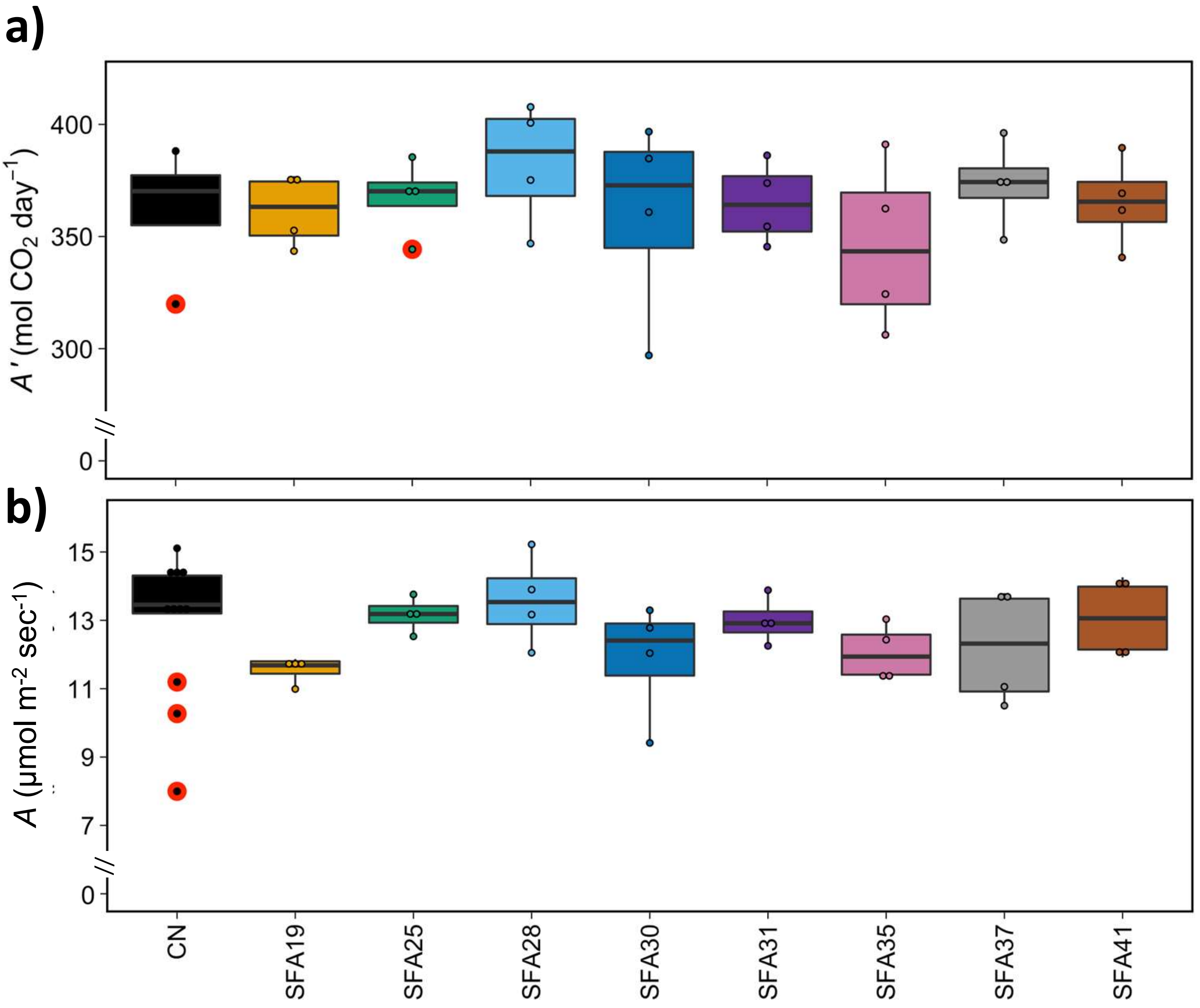

Supplemental Figure S7. a) Accumulated assimilation of CO_2 (*A'*) based on diurnal analysis of photosynthesis. **b)** Midday CO_2 assimilation. Measurements were made at ambient CO_2 and temperature on mature leaves. n=4 transgenics, n=13 for CNs. Linear mixed model analysis –lmer- accounting for block as random effect was used to compare genotypes; no significant differences were found. In boxplots: the box represents the middle 50% of the data (1st to 3rd quartile, Q1–Q3); the mid-line indicates the median (Q2); whiskers extend to the most extreme data points within 1.5× the interquartile range (IQR); and outliers beyond this range are shown as red dots.

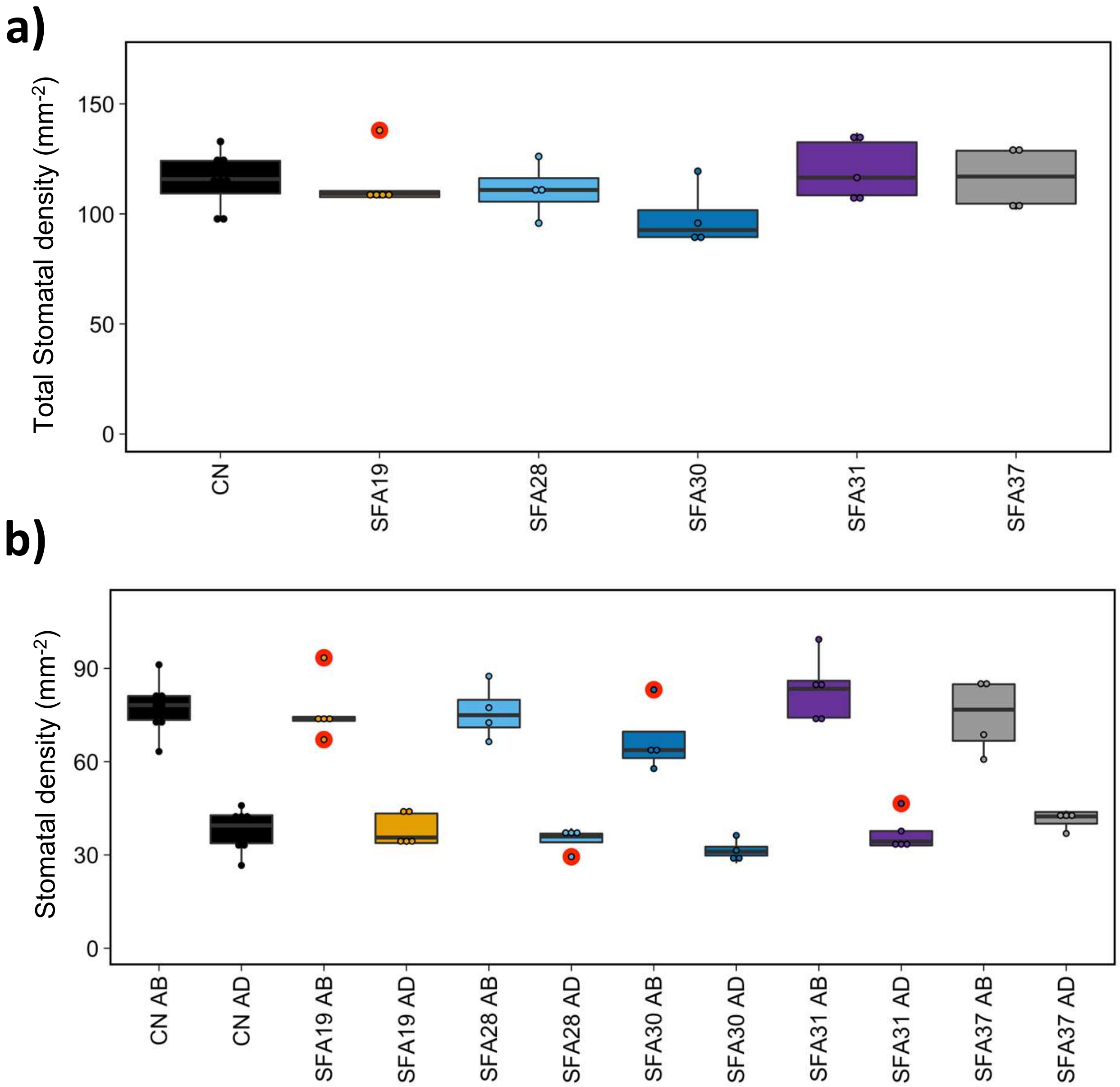

Supplemental Figure S8. SFA overexpression does not lead to significant differences in stomatal density. **a)** total stomatal density. **b)** Stomatal density in Adaxial (AD) and Abaxial (AB) surfaces of the leaf. Stomata counted from mature leaves after gas exchange measurements, n=4-8. Analysis of variance (aov) was used to compare stomatal densities between genotypes; no significant differences were found. In boxplots: the box represents the middle 50% of the data (1st to 3rd quartile, Q1–Q3); the mid-line indicates the median (Q2); whiskers extend to the most extreme data points within 1.5× the interquartile range (IQR); and outliers beyond this range are shown as red dots.

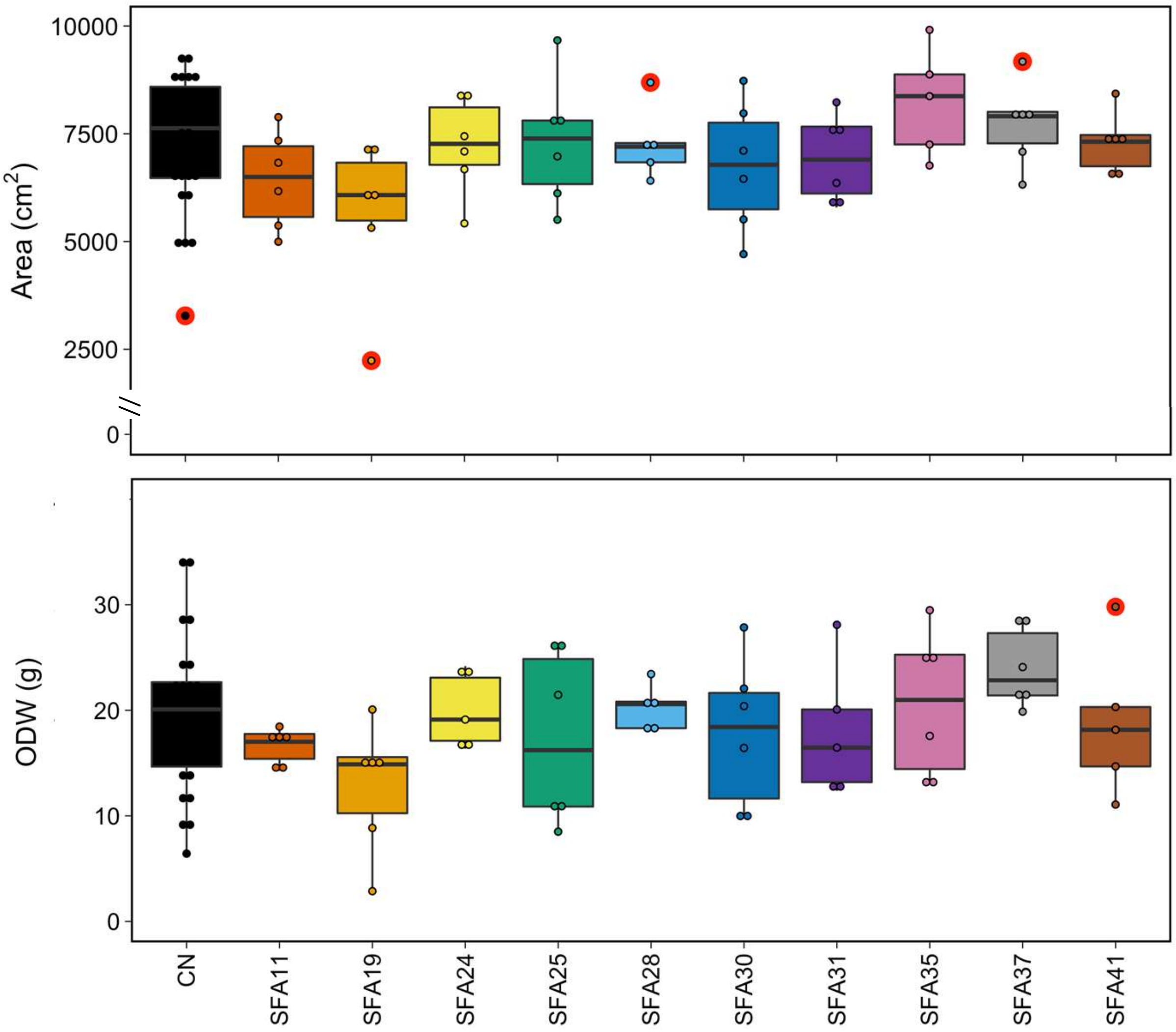

Supplemental Figure S9. Simultaneous increases in SBPase, FBP Aldolase and AGPase do not affect biomass in glasshouse-grown plants at the onset of flowering (56 days after sowing) in any of the lines studied. n= 6 for each SFA line, n=9 for CN. Leaf area (Area) at the top and Total over-ground dry weight (ODW) at the bottom. Linear mixed model analysis –lmer- accounting for block as random effect was used to compare genotypes. No significant differences were found. In boxplots: the box represents the middle 50% of the data (1st to 3rd quartile, Q1–Q3); the mid-line indicates the median (Q2); whiskers extend to the most extreme data points within 1.5× the interquartile range (IQR); and outliers beyond this range are shown as red dots.

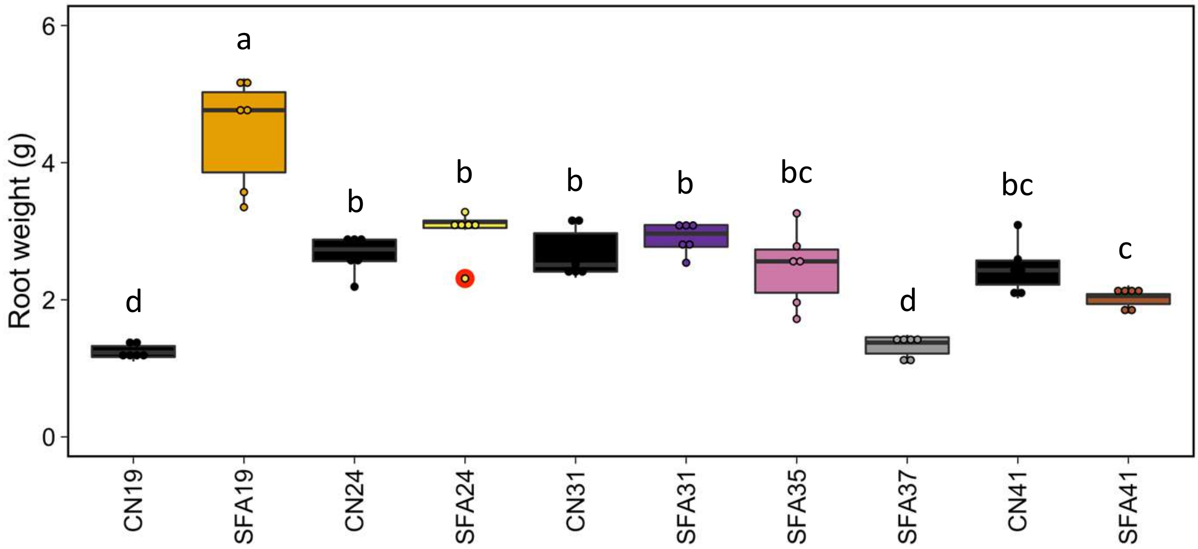

Supplemental Figure S10. SFA overexpression does not lead to a consistent change in root biomass. Root biomass was measured 37 days after sowing (n = 6). Analysis of variance (ANOVA) followed by Tukey's HSD test was used to assess statistical differences between genotypes. Different letters above boxplots indicate statistically significant differences ($P < 0.05$). In boxplots: the box represents the interquartile range (IQR; Q1–Q3), the line inside the box marks the median (Q2), whiskers extend to the most extreme data points within 1.5× IQR, and individual points represent biological replicates.
